# The STEAP4 target NQO1 mediates colon tumorigenesis

**DOI:** 10.1242/jcs.263402

**Published:** 2025-05-22

**Authors:** Kunlun Yin, Luke Villareal, Xiangxiang Wu, Mariella Arcos, Jordan Lee, David R. Martin, Julie G. In, Kimberly Leslie, Donna D. Zhang, Xiang Xue

**Affiliations:** ^1^ Department of Biochemistry and Molecular Biology, University of New Mexico, Albuquerque, NM 87131, USA; ^2^ Division of Molecular Medicine, Department of Internal Medicine, University of New Mexico, Albuquerque, NM 87131, USA; ^3^ Department of Pathology, University of New Mexico, Albuquerque, NM 87131, USA; ^4^ Division of Gastroenterology, Department of Internal Medicine, University of New Mexico, Albuquerque, NM 87131, USA; ^5^ Center for Inflammation Science and Systems Medicine, The Herbert Wertheim UF Scripps Institute for Biomedical Innovation and Technology, Jupiter, FL 33458, USA

**Keywords:** Colorectal cancer, Iron, NQO1, STEAP4, NRF2

## Abstract

Colorectal cancer (CRC) remains a major global health concern, necessitating advancements in therapeutic strategies. Understanding the mechanisms driving CRC is crucial for developing effective treatments. Previous studies, including our own, highlight the role of six-transmembrane epithelial antigen of prostate 4 (STEAP4) in promoting colon tumorigenesis through reactive oxygen species (ROS) generation, making it a promising target. Our research provides compelling evidence that STEAP4 knockout significantly reduces colon tumorigenesis in a genetically engineered mouse model. Suppressing STEAP4 via knockdown techniques effectively attenuated the nuclear factor erythroid 2-related factor 2 (NRF2)–NAD(*P*)H:quinone oxidoreductase 1 (NQO1) signaling pathway, inducing apoptosis and autophagy, leading to substantial reductions in xenograft tumor growth. In contrast, STEAP4 overexpression amplified ROS production and activated the NRF2–NQO1 pathway in a ferric iron (Fe^3+^)-dependent manner. Notably, bioactivatable drugs targeting NQO1 were highly effective at eradicating STEAP4-overexpressing colon cancer cells. These findings highlight the potential of targeted therapeutic interventions for CRC, particularly through STEAP4 modulation. In conclusion, our study advances understanding of the role of STEAP4 in colon tumorigenesis, offering promising avenues for novel CRC treatments.

## Teaser

**Summary:** Our study reveals the protumorigenic role of STEAP4 in colorectal cancer, highlighting its impact on the NRF2–NQO1 pathway, reactive oxygen species production and the therapeutic potential of NQO1-targeting drugs.

## INTRODUCTION

Colorectal cancer (CRC) stands as a formidable challenge in global healthcare, ranking among the leading causes of cancer-related mortality worldwide (Sung et al., 2021) and securing its position as the third most prevalent cancer in the United States (Siegel et al., 2022). Recognizing the urgency to develop effective treatments, this study centers its focus on unraveling the intricate mechanisms of colon cancer, crucial for the advancement of therapeutic interventions.

The human six-transmembrane epithelial antigen of prostate (STEAP) family, consisting of STEAP1–4, functions as unique mammalian metalloreductases, with roles in essential biological processes, including molecular trafficking, cell proliferation and apoptosis control (Gomes et al., 2012). STEAP transmembrane domains contain a single b-type heme, and a high-affinity flavin adenine dinucleotide (FAD) binding site coordinates intrasubunit, transmembrane electron transfer (Kleven et al., 2015). Although all four STEAP proteins promote the reduction of iron and copper *in vitro*, STEAP4 has the highest activity (Ohgami et al., 2006). Genome-wide association studies have linked various variants in *STEAP4* with the development of metabolic disorders (Scarl et al., 2017; Zhang et al., 2013). Several studies have shown that the expression of STEAP4 is modulated by inflammatory cytokines, hormones and other indicators of cellular stress, suggesting its role in maintaining normal metabolic function (Wellen et al., 2007; Liang et al., 2017).

STEAPs are also highly upregulated in several types of cancer, including CRC (Hubert et al., 1999; Grunewald et al., 2012; Xue et al., 2017), making them potential therapeutic targets. Previous studies have highlighted the pivotal role of STEAP4 in CRC using genetic mouse models. Intestinal-specific STEAP4 overexpression in mice is associated with increased mitochondrial iron accumulation, heightened oxidative stress-responsive protein NAD(*P*)H quinone dehydrogenase 1 (NQO1) and augmented susceptibility to colon tumors (Xue et al., 2017). Notably, elevated STEAP4 expression correlates with poor patient survival and is implicated in fostering colon tumorigenesis through enhanced Cu^2+^ uptake (Liao et al., 2020). However, specific pharmacological inhibitors of STEAP4 are lacking. Therefore, the present study explores alternative avenues, targeting downstream signaling pathways of STEAP4 such as NQO1.

NQO1 is an antioxidant enzyme associated with poor prognosis in various human cancers, including CRC. NQO1 knockdown in human CRC cell lines suppresses tumor growth (Oh et al., 2016). Recently, researchers have leveraged the ability of NQO1 to catalyze quinone drugs to produce long-lived, cell membrane-permeable hydrogen peroxide (H_2_O_2_) (Silvers et al., 2017). The tumor-specific loss of catalase amplifies H_2_O_2_ production (Doskey et al., 2016), leading to oxidative DNA lesions and selective tumor cell death (Huang et al., 2012).

The current research findings reveal that STEAP4 deficiency acts as a protective factor against colitis-associated colon cancer in mice, demonstrated by reduced xenograft tumor size and weight upon STEAP4 knockdown. Conversely, STEAP4 overexpression exacerbates reactive oxygen species (ROS) production and ferric iron (Fe^3+^)-dependent induction of NQO1. Notably, NQO1 bioactivatable quinone drugs, specifically β-lapachone (β-LPC) and KP372-18 (Jiao et al., 2022), outperform the conventional 5-fluorouracil (5-FU) in terms of killing efficiency in STEAP4-overexpressing colon tumor cells. This is of special relevance because a phase II clinical trial demonstrated β-LPC efficacy in a combination chemotherapy against pancreatic cancer (Khong et al., 2007).

In summary, targeting STEAP4 emerges as a feasible and promising strategy to inhibit colon tumorigenesis, offering potential avenues to overcome drug resistance. This research provides valuable insights into the intricate interplay of molecular pathways in colon cancer and lays the groundwork for the development of targeted therapies with the potential to revolutionize the treatment landscape for patients with CRC.

## RESULTS

### STEAP4 deficiency attenuates colon tumorigenesis in a mouse model with monoallelic *Apc* gene deletion

Adenomatous polyposis coli (*APC*) is a tumor suppressor and gatekeeper gene mutated in more than 80% of people with early-stage sporadic CRC. *Apc*^min/+^ mice develop more than 100 tumors in the small intestine (Xue et al., 2012), a phenomenon that is quite rare in the clinic. This was a major reason why we discontinued using *Apc*^min/+^ mice in our laboratory. The use of caudal type homeobox 2 (*Cdx2*) enables colon-specific gene targeting. The Cre–ERT2 fusion protein, which consists of Cre recombinase fused to a triple mutant form of the human estrogen receptor, does not bind its natural ligand (17β-estradiol) at physiological concentrations but will bind synthetic estrogen receptor ligands such as 4-hydroxytamoxifen. Mice with monoallelic disruption of *Apc* specifically in the colon develop a robust increase in colon adenomatous polyps within 3 months, and dextran sodium sulfate (DSS)-induced inflammation accelerates tumor growth to less than 1.5 months (Xue et al., 2016; Yin et al., 2021; Arcos et al., 2024). Additionally, Cre–ERT2 STEAP4 knockout mice (Liao et al., 2020) and villin–STEAP4-overexpressing mice (Xue et al., 2017) treated with the azoxymethane–DSS protocol have been published. Therefore, we employed a tamoxifen and DSS protocol on the *Steap4*^F/F^ *Cdx2*^Cre-ERT2^ *Apc*^F/+^ mouse model to induce colitis-associated colon cancer, as previously described (Yin et al., 2021).

**Fig. 1.**
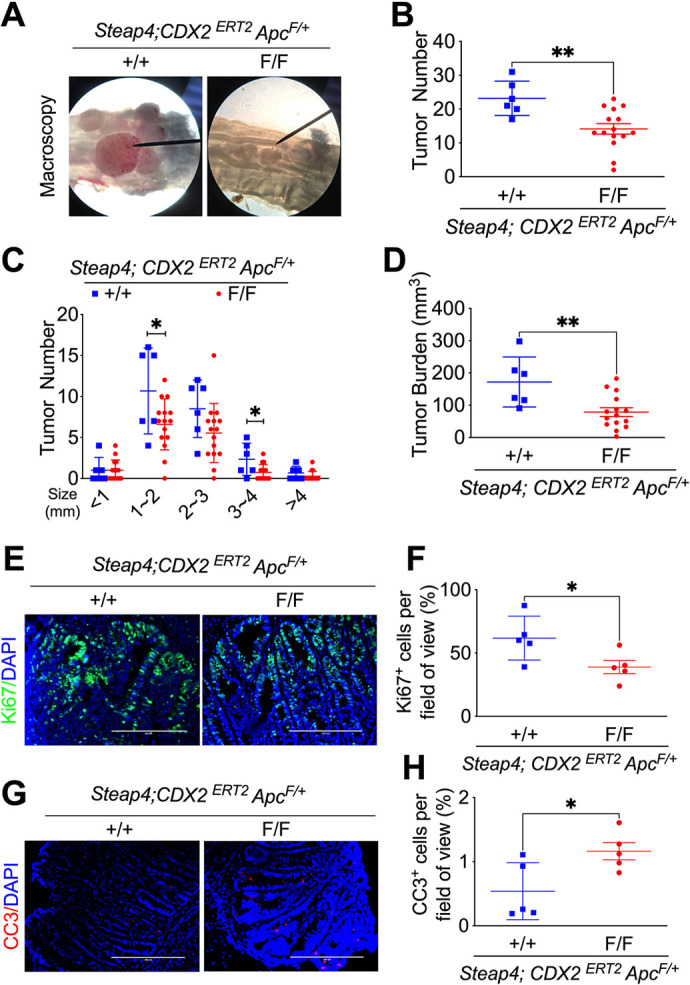
**Colon epithelial cell-specific *Steap4* knockout suppresses mouse colon tumor development.** (A–D) Macroscopic images of colon tumors (A), total tumor count (B), tumor distribution according to different sizes (C) and overall tumor burden (D) are presented for *Steap4* wild-type (*N*=6) and knockout (*N*=15) mice. Images in A are representative of at least two independent experiments. (E,F) Representative images of Ki67 staining (E) and its quantification (F) show differences in proliferative activity between *Steap4* wild-type (*N*=5) and knockout (*N*=5) mice. Images in E are representative of two experiments. (G,H) Representative images (G) and quantification (H) of CC3 staining for tumors in *Steap4* wild-type (*N*=5) and knockout (*N*=5) mice provide insights into apoptotic processes. Images in G are representative of two experiments. *P<0.05, **P<0.01. Unpaired two-tailed *t*-tests were applied for data in B, D, F and H; data in C underwent analysis through two-way ANOVA. Scale bars: 200 μm. Graphs show mean±s.e.m. with individual data points.

*Steap4* knockout mice exhibited significantly longer colon lengths compared to *Steap4* wild-type mice (Fig. S1A,B). Notably, STEAP4 deficiency resulted in decreased gross tumor growth (Fig. 1A), reduced tumor number (Fig. 1B), fewer tumors at specific sizes (1–2 mm and 3–4 mm; Fig. 1C) and diminished tumor burden (Fig. 1D). Quantitative PCR (qPCR) analysis revealed a significant decrease in *Steap4* expression in normal and tumor colon tissues of *Steap4* knockout mice compared to those of *Steap4* wild-type mice (Fig. S1C). Hematoxylin and Eosin (H&E) staining demonstrated a notable reduction in the percentage of low-grade adenoma after STEAP4 depletion (Fig. S1D,E). Furthermore, Ki67 (also known as MKI67) staining (Fig. 1E,F) and cleaved caspase-3 (CC3) staining (Fig. 1G,H) revealed that STEAP4 deficiency suppressed cell proliferation and enhanced cell death. These findings collectively indicate that STEAP4 deficiency effectively mitigates colon tumor development.

### STEAP4 knockdown suppresses colon tumor cell growth *in vitro* and *in vivo*

**Fig. 2.**
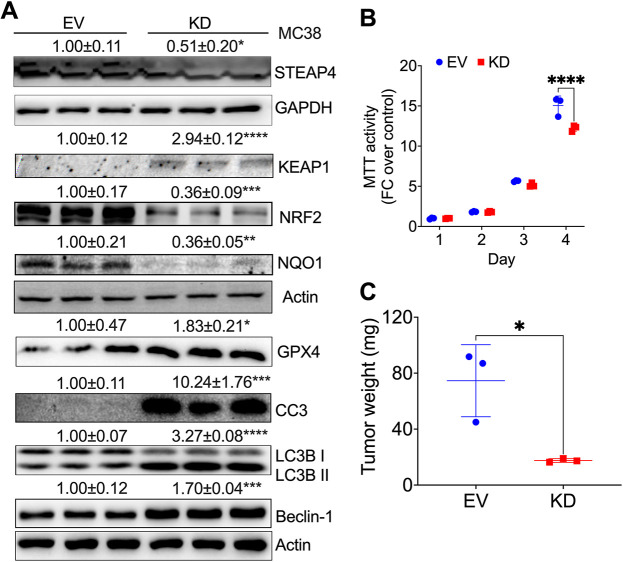
***Steap4* knockdown suppresses mouse colon tumor cell growth *in vitro* and *in vivo*.** (A) Western blot analysis provides insights into *Steap4* knockdown (KD) efficiency in MC38 cells compared to the control empty vector (EV). (B) Impact of *Steap4* KD on cell viability, as determined by MTT assay. FC, fold change. (C) Xenograft tumor weights for MC38 cells with *Steap4* knockdown or control EV are presented. *P<0.05, **P<0.01, ***P<0.001, ****P<0.0001. Two-way ANOVA was applied for data in B; unpaired two-tailed *t*-tests were used for data in C. *N*=3. Graphs show mean±s.e.m. with individual data points.

To investigate the impact of reduced STEAP4 expression on colon tumorigenesis, we assessed the effects of *Steap4* knockdown in murine MC38 CRC cells. Stable *Steap4* knockdown in MC38 cells was achieved through virus infection, validated by western blot analysis (Fig. 2A). Given that downregulation of STEAP4 reduces ROS levels in preosteoclasts (Zhou et al., 2013), we examined the KEAP1–NRF2–NQO1 pathway, a master antioxidant system regulating cellular responses to ROS. Following *Steap4* knockdown, we observed increased Kelch-like ECH-associated protein 1 (KEAP1) expression but decreased levels of nuclear factor erythroid 2-related factor 2 (NRF2; also known as NFE2L2) and its targets NQO1 and heme oxygenase-1 (HO-1; also known as HMOX1) (Tsai et al., 2022) (Fig. 2A). qPCR analysis revealed decreased expression of antioxidant genes, including *Nrf2*, *Nqo1* and *Gpx4*, whereas the stress-inducible antioxidant protein sestrin 2 (*Sesn2*) remained unchanged (Fig. S2A–D). Examination of key iron metabolic genes – such as ferritin heavy chain 1 (*Fth1*), transferrin receptor (*Tfrc*), nuclear receptor coactivator 4 (*Ncoa4*) and divalent metal transporter 1 (*Dmt1*; also known as *Slc11a2*) – showed decreased mRNA levels (Fig. S2E–H). Also, we found downregulation of the cell proliferation marker gene proliferating cell nuclear antigen (*Pcna*), the cell cycle gene cyclin D1 (*Ccnd1*) and *Steap4* (Fig. S2I–K).

Ferroptosis is a form of regulated cell death that is initiated by excessive iron and lipid peroxidation (Zhang et al., 2023). However, the ferroptosis inhibitor GPX4 was increased after STEAP4 knockdown at the protein level (Fig. 2A). ROS also play a crucial role in the regulation of various forms of cell death, including autophagy and apoptosis (Shang et al., 2021). Interestingly, STEAP4 deficiency increased the expression of the apoptotic marker CC3, the ratio of autophagy marker LC3 (also known as MAP1LC3)II to LC3I, and the expression of autophagy-related protein beclin-1 (Fig. 2A). Consistent with these molecular changes, STEAP4 deficiency suppressed colon tumor cell growth *in vitro* (Fig. 2B) and xenograft tumor growth *in vivo* (Fig. 2C). Collectively, these findings suggest that reducing STEAP4 expression is effective in modulating ROS homeostasis, activating non-ferroptotic cell death signaling pathways and suppressing colon tumor cell growth both *in vitro* and *in vivo*.

### STEAP4 overexpression activates the NRF2–NQO1 pathway in human CRC cells

**Fig. 3.**
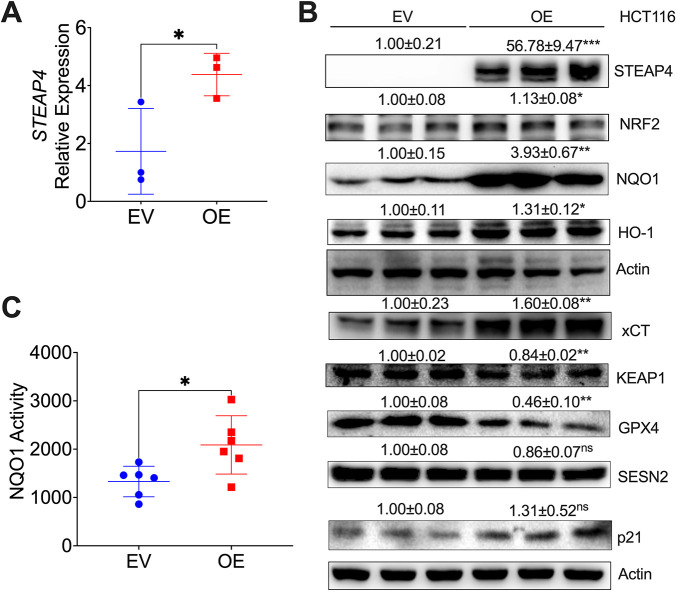
**STEAP4 overexpression in human HCT116 cells activates the NRF2 signaling pathway.** (A–C) Results from qPCR analysis of *STEAP4* (*N*=3) (A), western blot analysis (*N*=3) (B) and luciferase assay for NQO1 activity (*N*=6) (C) illustrate the impact of STEAP4 overexpression (OE) in HCT116 cells compared to the control EV. *P<0.05, **P<0.01, ****P<0.0001; ns, not significant. Unpaired two-tailed *t*-tests were employed for data in A and C. Graphs show mean±s.e.m. with individual data points.

To mimic STEAP4 function in people with CRC, we overexpressed STEAP4 in the HCT116 cell line. We chose HCT116 cells because these cells have very low baseline levels of STEAP4 compared to other CRC cell lines, whereas the normal human colon cell line NCM460 shows undetectable STEAP4 expression (Fig. S3A). qPCR data showed increased expression of *STEAP4*, *NRF2* and *NQO1*, and *SESN2* remained unchanged (Fig. 3A; Fig. S3B–D). Key iron metabolic genes, such as *FTH1* and *NCOA4*, showed increased mRNA levels (Fig. S3E,F). Also, we found upregulation of *PCNA* (Fig. S3G). Immunoblot analysis demonstrated that STEAP4 protein, and the antioxidant proteins NRF2, NQO1, HO-1 and solute carrier family 7 member 11 (SLC7A11; also commonly known as xCT) were increased, whereas KEAP1 and GPX4 were decreased, after STEAP4 overexpression (Fig. 3B). SESN2 and the cell cycle regulation protein p21 (also known as CDKN1A) were not altered in these cells (Fig. 3B). Luciferase assay revealed that NQO1 activity was increased in the STEAP4 overexpression cell line (Fig. 3C). These data indicate that STEAP4 overexpression activates the antioxidant NRF2–NQO1 pathway in HCT116 cells.

### Oxidative stress response during STEAP4-catalyzed Fe^3+^ reduction *in vitro*

**Fig. 4.**
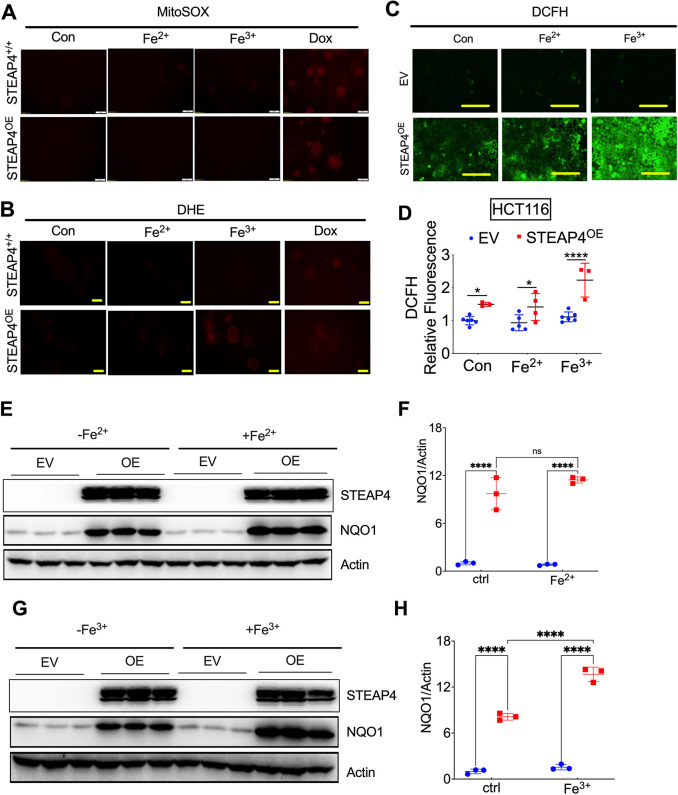
**Oxidative stress response during STEAP4-catalyzed Fe^3+^ reduction *in vitro*.** (A,B) Representative images for MitoSOX assay (A) and dihydroethidium (DHE) staining (B) in mouse colon enteroid cultures derived from *STEAP4*^OE^ mice and their littermates (*STEAP4*^+/+^). Images are representative of two experiments. (C,D) Representative images (C) and quantitative analysis (D) of 2′,7′-dichlorofluorescein diacetate (DCFH-DA) staining in human colon-derived HCT116 cancer cells with (*N*=6) or without (*N*=3) STEAP4 overexpression. Images in C are representative of two experiments. (E,F) Immunoblot analysis (E) and quantification (F) for HCT116 cells treated with Fe^2+^ (*N*=3). (G,H) Immunoblot analysis (G) and quantification (H) for HCT116 cells treated with Fe^3+^ (*N*=3). Enteroids or HCT116 cells were treated with 10 μM doxorubicin (Dox), 100 μM ferrous sulfate (Fe^2+^) or 100 μM ferric chloride (Fe^3+^) overnight. ns, not significant; *P<0.05, ****P<0.0001. Data in D, F and H were analyzed with two-way ANOVA. Scale bars: 200 μm. Graphs show mean±s.e.m. with individual data points.

Our earlier findings revealed dysregulation in mitochondrial iron balance, enhanced ROS production and increased susceptibility to mouse models of colitis and CRC when STEAP4 is overexpressed in intestinal epithelial cells (Xue et al., 2017). We hypothesized that ROS are generated during the process of STEAP4-catalyzed reduction of Fe^3+^ to Fe^2+^ in the mitochondria. To test this hypothesis, we established enteroid lines from mice with intestinal-specific overexpression of human STEAP4–GFP (*STEAP4*^OE^) and their littermates (Fig. S4). The MitoSOX™ Red reagent, a mitochondrial superoxide indicator, indicated that mitochondrial superoxide levels were not increased by STEAP4 overexpression, regardless of treatment with ferrous sulfate (FS; Fe^2+^) or ferric chloride (FC; Fe^3+^). In contrast, the positive control doxorubicin (Dox) significantly increased mitochondrial superoxide levels (Fig. 4A).

Dihydroethidium (DHE) staining, specific for superoxide and H_2_O_2_, showed that Fe^3+^ treatment significantly increased the signal in *STEAP4*^OE^ enteroids compared to the wild-type control group, whereas Fe^2+^ treatment did not elicit a similar response. The positive control Dox increased the signal in both genotypes (Fig. 4B). These data suggest that H_2_O_2_ is the major type of ROS produced by STEAP4. Indeed, 2′,7′-dichlorofluorescein diacetate (DCFH-DA) staining in colon-derived HCT116 cells showed that H_2_O_2_ levels were significantly increased after STEAP4 overexpression, and the addition of Fe^3+^, but not Fe^2+^, further increased H_2_O_2_ levels (Fig. 4C,D). Consistently, western blot analysis demonstrated that NQO1 expression was potentiated by Fe^3+^, but not Fe^2+^, in STEAP4 overexpression cells (Fig. 4E–H). Given that STEAP4 overexpression increases NQO1 protein and H_2_O_2_ levels simultaneously, we tested whether H_2_O_2_ can elevate NQO1 expression. Western blot results confirmed that H_2_O_2_ can increase NQO1 in a concentration-dependent manner (Fig. S5A,B). In conclusion, our study confirmed that H_2_O_2_, not superoxide, is formed during the process of STEAP4-catalyzed reduction of Fe^3+^ to Fe^2+^ in mitochondria.

### STEAP4 overexpression increases cell sensitivity to NQO1 bioactivatable drugs

Fluorouracil (5-FU) is a first-line chemotherapy for CRC in the clinic, and our results indicate that HCT116 cells overexpressing STEAP4 are resistant to 5-FU treatment (Fig. S6A). Therefore, it is important to identify alternative therapeutic targets for patients with CRC with elevated STEAP4 expression. Our data show a positive correlation between NRF2 and NQO1 expression with STEAP4 expression in our cell lines (Fig. 2A, Fig. 3B and Fig. 4E,G). By mining gene expression data from TNMPlot (https://tnmplot.com/analysis2/; Bartha and Győrffy, 2021), we observed increased *NQO1* expression (Fig. S6B), but decreased *STEAP4* expression (Fig. S6C), in colon tumors compared to normal colon tissues. Moreover, *NQO1* mRNA expression negatively correlated with *STEAP4* mRNA expression in colon tumor tissues (Fig. S6D). This decrease in *STEAP4* expression in colon tumors contrasts with our previous findings (Xue et al., 2017), highlighting the complexity of the relationship between STEAP4 and NQO1 and the need for further investigation.

**Fig. 5.**
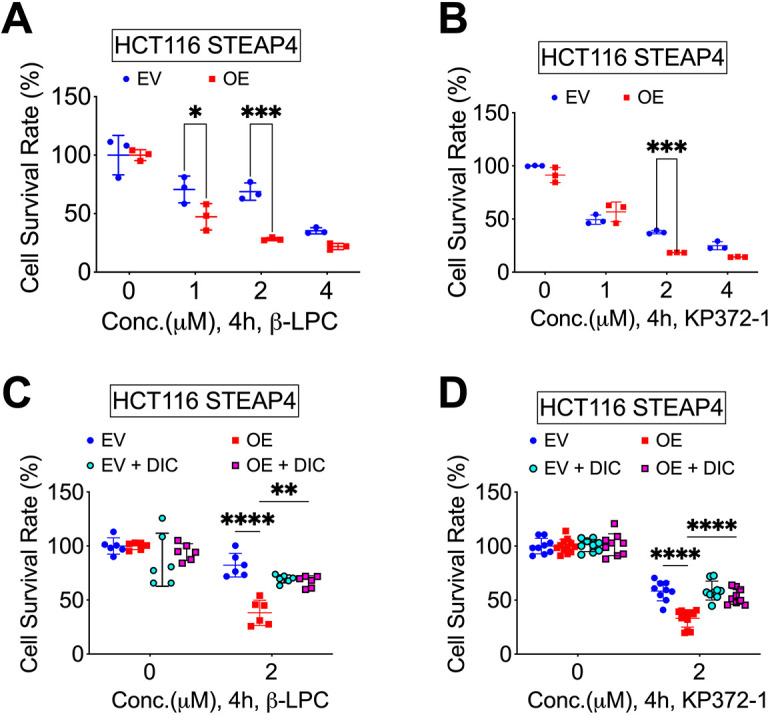
**STEAP4 overexpression increases oxidative stress and susceptibility to NQO1 bioactivatable drugs in colorectal cancer.** (A–D) Cell survival, determined by MTT assay 24 h following treatment with NQO1 bioactivatable drug β-lapachone (β-LPC) (*N*=3) (A), NQO1 bioactivatable drug KP-372 (*N*=3) (B), β-LPC and NQO1 inhibitor dicoumarol (DIC) (*N*=6) (C), or KP-372 and DIC (*N*=9) (D) for 4 h in HCT116 cells with stable STEAP4 OE or control EV. *P<0.05, **P<0.01, ***P<0.001, ****P<0.0001. Two-way ANOVA was applied. Graphs show mean±s.e.m. with individual data points.

Analysis of a CRC dataset from Oncomine further revealed that high NQO1 expression is significantly associated with poor survival in The Cancer Genome Atlas dataset (*n*=21 NQO1 high, *n*=24 NQO1 low; *P*=0.0001, log-rank analysis) (Oh et al., 2016). Notably, STEAP4-overexpressing cells were more sensitive than empty vector (EV)-transfected parental HCT116 cells to both β-LPC and KP372-1 treatments (Fig. 5A,B). Furthermore, dicoumarol, an NQO1 inhibitor (Asher et al., 2006), rescued the cell death induced by β-LPC and KP372-1 (Fig. 5C,D). Taken together, these findings suggest that STEAP4 overexpression increases oxidative stress and enhances susceptibility to NQO1 bioactivatable drugs in CRC.

## DISCUSSION

STEAPs are associated with metabolic diseases and are overexpressed in several human cancers (Korkmaz et al., 2005), underlining their physiological function in maintaining cellular iron homeostasis. Our investigation into the role of STEAP4 in colon cancer has yielded significant findings, with broad implications for understanding and potentially treating this malignancy. Our primary discovery involves the observation that STEAP4, found to be upregulated in various cancer types (Jin et al., 2015), plays a crucial role in colon cancer. This insight into the molecular mechanisms associated with STEAP4 could pave the way for new therapeutic interventions.

In our study, mice with colon epithelium-specific *Steap4* knockout displayed a notable reduction in both the number and size of tumors. This establishes STEAP4 as a key contributor to tumorigenesis in the colon. The impact of STEAP4 on tumor growth was further validated in xenograft models, in which STEAP4 knockdown led to significant suppression of tumor growth. These findings collectively underscore the potential therapeutic relevance of targeting STEAP4 in the context of colon cancer.

**Fig. 6.**
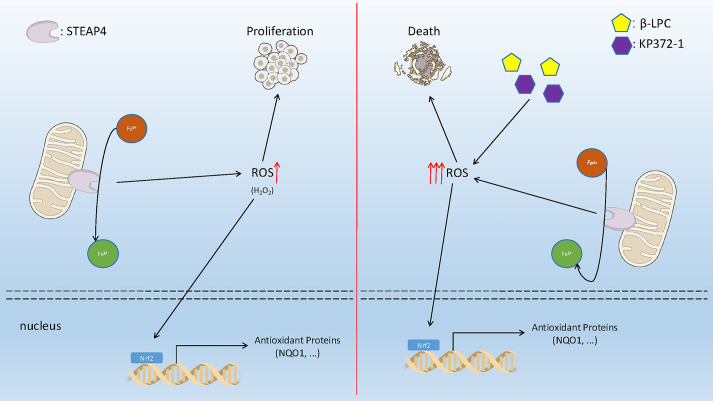
**A proposed working model.** (A) STEAP4, a mitochondrial protein, functions as a catalyst for the reduction of Fe^3+^ into Fe^2+^ while generating H_2_O_2_. Both Fe^2+^ and H_2_O_2_ contribute to STEAP4-mediated colon cancer cell proliferation. Antioxidant proteins including NQO1 are induced in STEAP4-overexpressing cells through NRF2 transcriptional regulation, acting to counteract the cytotoxic effects of H_2_O_2_. (B) Capitalizing on the characteristic of tumors with low catalase expression, NQO1 bioactivatable drugs (β-LPC and KP372-1) are strategically employed to generate an overwhelming amount of H_2_O_2_. This approach aims to selectively target and eliminate colon cancer cells characterized by high expression of STEAP4. Figure made using public domain icons from https://bioart.niaid.nih.gov/.

Our mechanistic investigations revealed intriguing connections between STEAP4 and the NRF2–NQO1 pathway (Fig. 6A). STEAP4 knockdown repressed this pathway in CRC cells, whereas its overexpression activated it. This dynamic regulation suggests a complex interplay between STEAP4 and oxidative stress responses, providing new insights into the molecular landscape of colon cancer. Moreover, our study uncovered that STEAP4 overexpression in colon cancer cells is associated with elevated levels of ROS. This elevation in ROS levels promotes cell proliferation and triggers an antioxidant response. Building upon this, our data demonstrated that treatment with NQO1 bioactivatable drugs, specifically β-LPC and KP372-1, induced robust oxidative stress response, leading to significant cell death (Fig. 6B). This highlights a potential therapeutic avenue for selectively targeting tumor cells with high STEAP4 expression.

Our study delves into the nuanced regulation of antioxidant proteins in response to STEAP4 levels. Notably, the expression levels of NQO1 and HO-1 were altered, suggesting a sophisticated cellular mechanism that fine-tunes antioxidant protein expression to counteract distinct oxidative stress stimuli. It is intriguing that the expression level of SESN2 was not affected by changes in STEAP4 levels. In a previous study, we demonstrated that hemin induces the simultaneous expression of NQO1, HO-1 and SESN2 (Kim et al., 2019). These findings suggest that cells have evolved an intricate mechanism capable of fine-tuning the expression levels of different antioxidant proteins to counteract various oxidative stress stimuli. This discovery adds a layer of complexity to our understanding of cellular responses to oxidative stress in the context of STEAP4. Furthermore, we explored the involvement of STEAP4 in autophagy, a cellular process with dual roles in both cell death and survival (Das et al., 2012). Unraveling how STEAP4 mediates autophagy in colon cancer cells could provide valuable insights into its multifaceted role in cellular processes.

Looking ahead, it is crucial to translate our findings into clinically relevant applications. Subsequent studies should evaluate the efficacy of NQO1 bioactivatable drugs, such as β-LPC and KP372-1, in selectively targeting tumor cells with high STEAP4 expression using patient-derived models and mouse models (Kim et al., 2023). Addressing dose-limiting toxicity associated with these drugs is imperative (Viera and Patidar, 2020), and innovative strategies should be explored. Recent research on the synergistic effects of β-LPC and mitophagy inhibition on endometrial cancer (Gong et al., 2023) suggests that combination therapy could be a feasible route. RNA interference (RNAi), a technology capable of downregulating gene expression levels, presents another promising avenue. Given that several RNAi drugs are either approved by the US Food and Drug Administration (FDA) or in clinical trials (Zhang et al., 2021), and we have recently achieved successful delivery of nucleic acids into prostate cancer cells using mesoporous silica nanoparticles (Maestas-Olguin et al., 2023), strategies for delivering *STEAP4* siRNA or antisense oligonucleotides to CRC cells should also be considered.

In conclusion, our study positions *STEAP4* as an oncogene driving colon tumorigenesis through intricate regulatory mechanisms involving oxidative stress, antioxidant pathways and autophagy. These findings not only contribute to our understanding of colon cancer biology but also open avenues for targeted interventions that could potentially overcome chemoresistance and improve patient outcomes.

## MATERIALS AND METHODS

### Animals

Animal studies were conducted to address specific research questions, adhering to the Institute of Laboratory Animal Resources guidelines, and approved by the Institutional Animal Care and Use Committee (IACUC) at the University of New Mexico Health Sciences Center (Protocol# HSC-18-200699, 20-201060-HSC), following the National Institutes of Health guide for the care and use of laboratory animals (NIH Publications No. 8023, revised 1978). Mice, encompassing both sexes, were housed in standard cages under a 12-h light–dark cycle, in a temperature-controlled environment, with *ad libitum* access to a standard chow diet and water.

Intestinal-specific overexpression of human STEAP4–GFP (*STEAP4*^OE^) mice was generated as previously described (Xue et al., 2017). *Steap4*^F/F^ mice were produced utilizing exon 2–3 of the mouse *Steap4* gene as the conditional knockout region at Cyagen (Santa Clara, CA) and crossed with *Cdx2*^Cre-ERT2^*Apc*^F/+^ mice to generate *Steap4*^F/F^ *Cdx2*^Cre-ERT2^*Apc*^F/+^ mice. In the colitis-associated CRC model, 6- to 8-week-old *Steap4*^F/F^ *Cdx2*^Cre-ERT2^*Apc*^F/+^ mice, along with *Cdx2*^Cre-ERT2^*Apc*^F/+^ mice, were treated with 100 mg/kg tamoxifen for 3 days via intraperitoneal injection. Seven days later, they received water containing 1.5% DSS for 7 days, followed by regular drinking water for 28 days before they were killed.

To assess the necessity for STEAP4 under immune-intact conditions in the subcutaneous xenograft study, murine MC38 cells with STEAP4 knockdown were trypsinized, resuspended in sterile 1× PBS, counted and diluted to a concentration of 1×10^7^ cells/ml. Subsequently, 100 μl containing 1×10^6^ total cells was injected into the flanks of C57BL/6 mice. After 2 weeks, mice were killed using CO_2_, and tumors were collected for analysis.

### Histology and immunofluorescence staining

Colon tissues were dissected, washed with 1× PBS and fixed overnight at room temperature in 10% neutral buffered formalin before being embedded in paraffin. Tissue sections (5 μm) underwent deparaffinization in xylene and rehydration through an ethanol gradient. Histological analysis employed H&E staining on paraffin sections, which were microscopically evaluated by a gastrointestinal pathologist in a masked manner. For the tumorigenesis model, tissue classification included tubular adenoma with low-grade (score 1) versus high-grade (score 2) dysplasia, with the involved lesion percentage estimated. The final pathological score was determined by multiplying the lesion percentage by the grade score. The scoring system we used has been described in several of our publications (Yin et al., 2021; Kim et al., 2023; Liu et al., 2023; Arcos et al., 2024). The percentage refers to the proportion of the adenoma lesion area relative to the total tissue area, which typically includes both adenomatous and normal colon tissue on the section slides.

Immunofluorescence analysis utilized primary antibodies against Ki67 (12202T, Cell Signaling Technology) and CC3 (9664, Cell Signaling Technology) at a 1:100 dilution. Secondary antibodies were applied at a 1:200 dilution, and nuclei were stained with 4′,6-diamidino-2-phenylindole (DAPI). Imaging and analysis were conducted using a fluorescence microscope (EVOS, Thermo Fisher Scientific).

### Cell culture

Murine MC38 syngeneic CRC cells were generously provided by Dr Weiping Zou from the University of Michigan, and authentication of this cell line was performed by American Type Culture Collection (ATCC) using mouse short tandem repeat profiling (137XV). The human colorectal carcinoma cell line HCT116 was obtained from ATCC (CCL-247). Mycoplasma contamination was ruled out using the MycoStrip™ Mycoplasma Detection Kit (rep-mys-10, InvivoGene). Cells were cultured at 37°C in 5% CO_2_, maintained in Dulbecco's modified Eagle medium (DMEM) supplemented with 10% fetal bovine serum, and 1% penicillin and streptomycin (VWR). STEAP4 knockdown was achieved in the MC38 cell line by transduction with lenti-GIPZ-*Steap4*-VSVG (V2LMM_13428, Horizon Discovery). The knockdown was confirmed by real-time qPCR and western blotting after selecting cells with 1 µg/ml puromycin for 1 week. STEAP4 overexpression using pIRES puro3 *STEAP4-GFP* plasmid was also established in the HCT116 cells.

### Mouse enteroid culture

Enteroids derived from the colon of *STEAP4*^OE^ mice and their littermate controls were cultured in Matrigel with advanced DMEM/F12 complete media. The media were supplemented with growth factors, including epidermal growth factor (EGF), R-spondin, WNT3A and noggin, following a previously described protocol (Xue and Shah, 2013). Enteroids were treated overnight with 10 μM Dox, 100 μM FS (Fe²^+^) or 100 μM FC (Fe³^+^).

### MitoSOX and DHE staining

Mouse enteroids, cultured in a 12-well plate, were treated with Fe^2+^ or Fe^3+^ overnight. The following day, MitoSOX (M36008, Thermo Fisher Scientific) or DHE (D11347, Thermo Fisher Scientific) fluorescent dyes were added, and the enteroids were incubated for 30 min at 37°C in a 5% CO_2_ environment. Following the incubation, the enteroids were washed three times with PBS and imaged using an Olympus BX53 Digital Upright Microscope equipped with the TRITC filter.

### DCFH-DA staining

HCT116 cells (2×10^5^ cells/well) were plated in a 24-well plate using the method described previously (Kim et al., 2019) and incubated overnight. The following day, media were removed, and cells were stained with 100 µl of a 10 µM DCFH-DA solution (20656, Cayman Chemical). The cells were then incubated for 30 min at 37°C in a 5% CO_2_ environment in the dark. Subsequently, the staining solution was removed, and cells were washed with PBS. Representative images were captured using an Invitrogen Auto Imaging System (Thermo Fisher Scientific), and fluorescence was measured at excitation/emission=485/530 nm in endpoint mode with a SpectraMax M2 Microplate Reader (Molecular Devices). The fluorescence intensity was normalized by protein concentration.

### MTT assay

MC38 cells with STEAP4 knockdown were seeded at a concentration of 4000 cells/ml in 12-well plates. Subsequently, 125 μl of 5 mg/ml 3-(4,5-dimethylthiazol-2-yl)-2,5-diphenyltetrazolium bromide (MTT; Sigma) was added to each well, and the plates were incubated for 30 min. Following the incubation, dimethyl sulfoxide was added, and absorbance was measured at 570 nm using a SpectraMax M2 Microplate Reader (Molecular Devices).

### Real-time qPCR analysis

RNA extraction was performed using a reagent kit (IB47602, IBI Scientific). Subsequently, qPCR analysis was conducted on a LightCycler 480 instrument (Roche Diagnostics). mRNA levels were quantified via qPCR with the primers listed in Table S1. Normalization of gene expression levels was achieved using 18S (also known as *Rn18s*) as the reference gene. Changes in expression levels were calculated by comparing to the control.

### Western blot analysis

Cells were lysed with radioimmunoprecipitation (RIPA) buffer and incubated on ice for 30 min. Subsequently, cell extracts were centrifuged for 15 min at full speed (15,000 ***g***, 4°C), and the supernatant was collected for protein concentration quantification using the Bradford assay. Equal amounts of 30 μg of protein were loaded for sodium dodecyl sulfate polyacrylamide gel electrophoresis (SDS-PAGE), alongside molecular mass markers. The gels were run for 1 h and 30 min at 100 V. Proteins within the gels were then transferred onto nitrocellulose membranes for 1 h at 100 V using the wet transfer method.

Subsequently, the membranes were blocked with 3% milk for 1 h before being incubated with primary antibodies at 4°C overnight. The primary antibodies used included anti-β-actin (sc-47778, Santa Cruz Biotechnology, RRID:AB_626632, 1:1000), anti-beclin-1 (3495, Cell Signaling Technology, RRID:AB_1903911, 1:1000), anti-CC3 (9664, Cell Signaling Technology, RRID:AB_2070042, 1:500), anti-GPX4 (sc-166570, Santa Cruz Biotechnology, RRID:AB_2112427, 1:1000), anti-HO-1 (sc-390991, Santa Cruz Biotechnology, RRID:AB_3668907, 1:1000), anti-KEAP1 (sc-5149, Santa Cruz Biotechnology, RRID:AB_2861131, 1:1000), anti-MAP1LC3A/B (12741, Cell Signaling Technology, RRID:AB_2617131, 1:1000), anti-NQO1 (sc-32793, Santa Cruz Biotechnology, RRID:AB_628036, 1:1000), anti-NRF2 (16396-1-AP, Proteintech, RRID:AB_2782956, 1:1000), anti-p21 (27296-1-AP, Proteintech, RRID:AB_2880834, 1:1000), anti-SESN2 (sc-393195, Santa Cruz Biotechnology, RRID:AB_3668906, 1:1000), anti-STEAP4 (11944-1-AP, Proteintech, RRID:AB_2197868, 1:1000; NB100-68162, Novus, RRID:AB_1110724, 1:1000) and anti-xCT (26864-1-AP, Proteintech, RRID:AB_2880661, 1:1000). Secondary antibodies against mouse IgG (7076, Cell Signaling Technology, RRID:AB_330924, 1:2000) or rabbit IgG (7074, Cell Signaling Technology, RRID:AB_2099233, 1:2000) were also used. The original uncropped images are shown in Fig. S7.

### Luciferase reporter gene assay

HCT116 wild-type and STEAP4 overexpression cells were transfected with the human *NQO1*-ARE TATA-Inr luciferase reporter plasmid (Lau et al., 2010). After 48 h of transfection, cells were lysed with lysis buffer, and the supernatant was collected. Luciferase activity was measured using a kit from Promega (E1500). Briefly, cells were seeded in a 24-well plate and incubated overnight at 37°C and 5% CO_2_. The following day, cells were rinsed with PBS, and PBS was removed before adding the lysis buffer. All solutions and cells were transferred to a new tube and placed on ice. After centrifugation at 12,000 ***g*** for 2 min at 4°C, the supernatant was collected. Twenty microliters of supernatant were transferred to a 96-well plate, and 100 µl Luciferase Assay Reagent (Promega) per well was added. Luciferase intensity was measured by a plate reader, and the activity was normalized by protein concentration.

### Statistical analysis

All *in vitro* experiments were performed in triplicate and independently repeated at least three times. The data are expressed as mean±s.e.m. with individual data points shown. Statistical analysis was carried out using independent unpaired two-tailed *t*-tests, one-way ANOVA or two-way ANOVA, as appropriate. *P*<0.05 was considered statistically significant.

### Declaration of generative AI and AI-assisted technologies in the writing process

During the preparation of this work, the authors used ChatGPT3.5 to improve language and readability. After using this tool/service, the authors reviewed and edited the content as needed and take full responsibility for the content of the publication.

## Supplementary Material

10.1242/joces.263402_sup1 Supplementary information
