# Supplementary material for: The STEAP4 target NQO1 mediates colon tumorigenesis
Source: J Cell Sci. 2025 May 22;138(10):jcs263402. doi: 10.1242/jcs.263402 (PMC12148032; doi:10.1242/jcs.263402)
Supplement: Supplementary information [file joces-138-263402-s1.pdf]

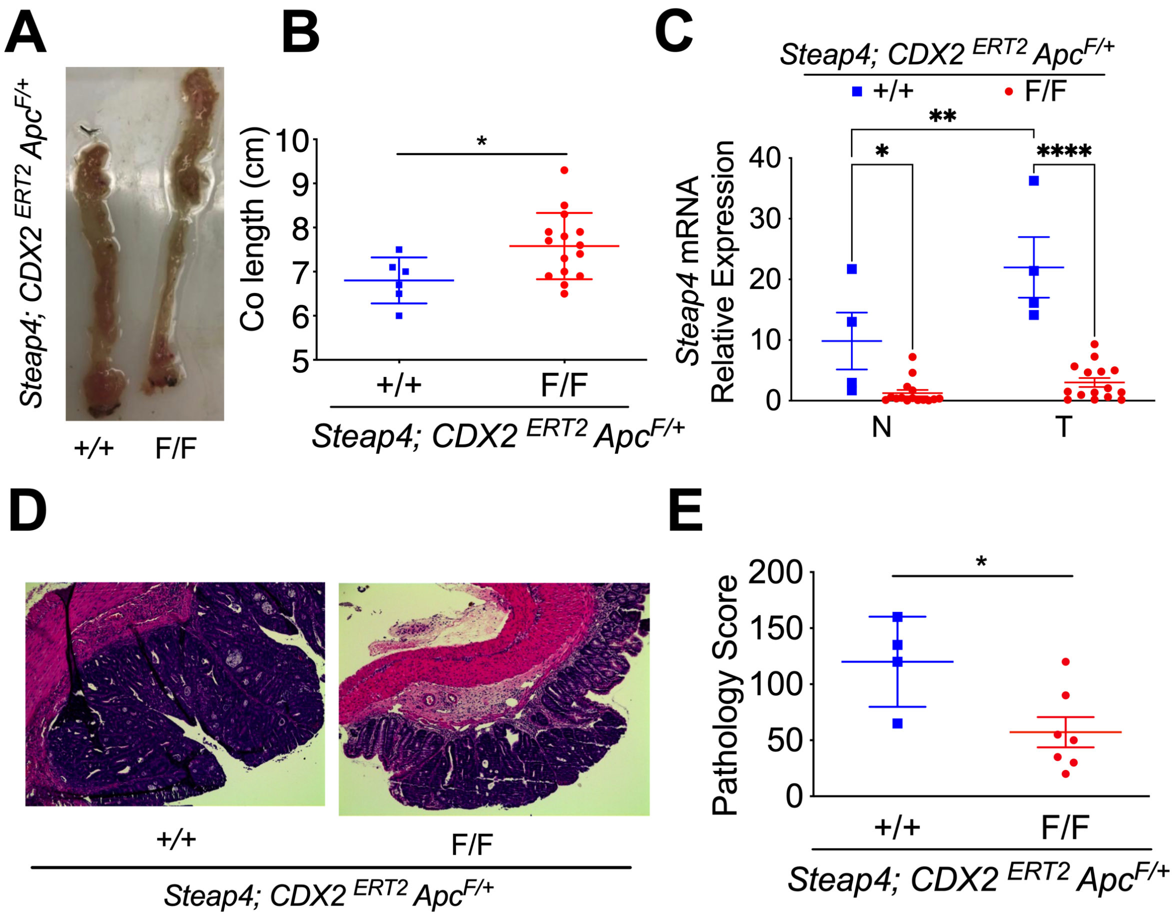

Fig. S1. Colon epithelial cell specific *Steap4* knockout mice have longer colon length and lower pathological score after a colon tumor development protocol.

(**A**) Representative gross images for colon tissues, (**B**) colon length, (**C**) qPCR analysis of *Steap4* expression in normal (N, N=4) and tumor (T, N=15) colons, (**D**) H&E staining and (**E**) histopathological scoring of colon tissues from *Steap4*$^{F/F}$; CDX2^{ERT2}Apc$^{F/+}$ (N=7) and *Steap4*$^{+/+}$; CDX2^{ERT2}Apc$^{F/+}$ (N=4) mice. *p<0.05, **p<0.01, ****p<0.0001. Data in B and E were analyzed with unpaired t-test. Data in D were analyzed with two-way ANOVA.

Journal of Cell Science • Supplementary information

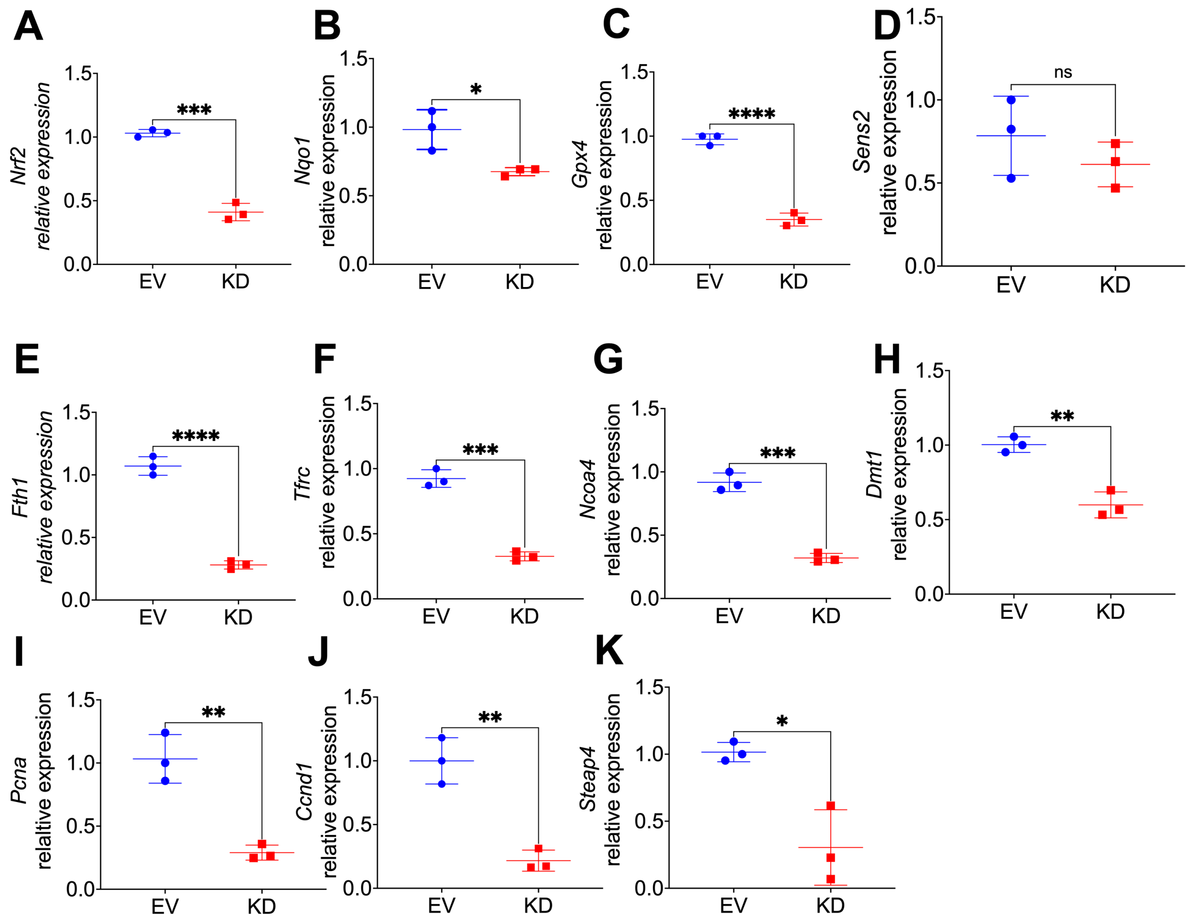

Fig. S2. qPCR analysis in MC38 cells with or without *Steap4* knockdown. A few representative genes involved in (**A-D**) anti-oxidation, (**E-H**) iron metabolism, (**I**) cell proliferation, (**J**) cell cycle and (**K**) *Steap4* were measured. ns, not significant, *p<0.05, **p<0.01, ***p<0.001, ****p<0.0001. Data were analyzed with unpaired t-test. N=3.

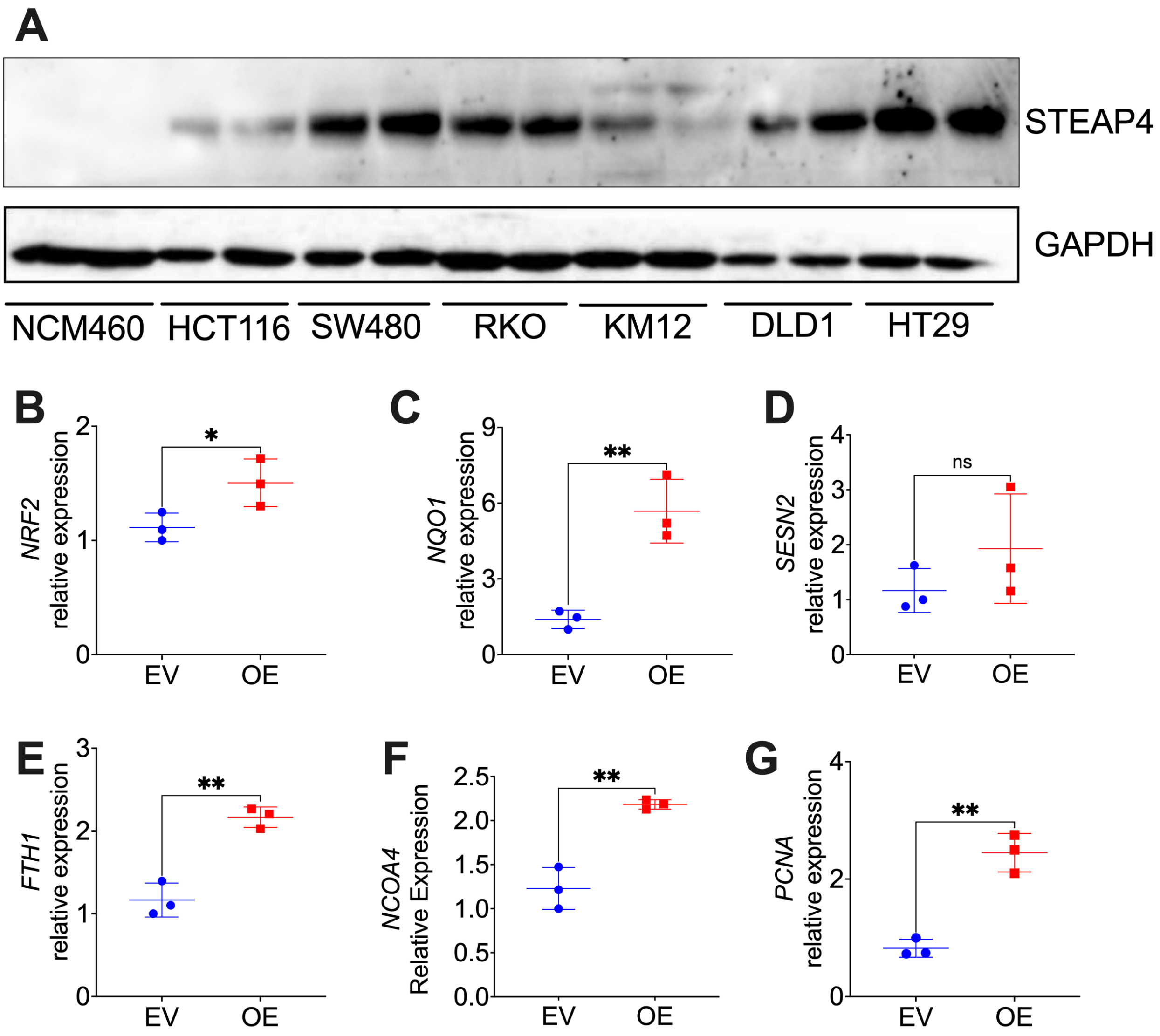

Fig. S3. STEAP4 protein expression is higher in colorectal cancer cells compared to normal cells and gene expression in HCT116 cells with *STEAP4* overexpression. (A) Western blot analysis (N=2) shows STEAP4 protein expression levels across multiple colorectal cancer and normal cell lines. qPCR analysis of genes involved in (**B-D**) anti-oxidation, (**E, F**) iron metabolism and (**G**) cell proliferation from HCT116 cells with STEAP4 overexpression (OE) or control empty vector (EV). ns, not significant, *p<0.05, **p<0.01. Data were analyzed with unpaired t-test. N=3.

Journal of Cell Science • Supplementary information

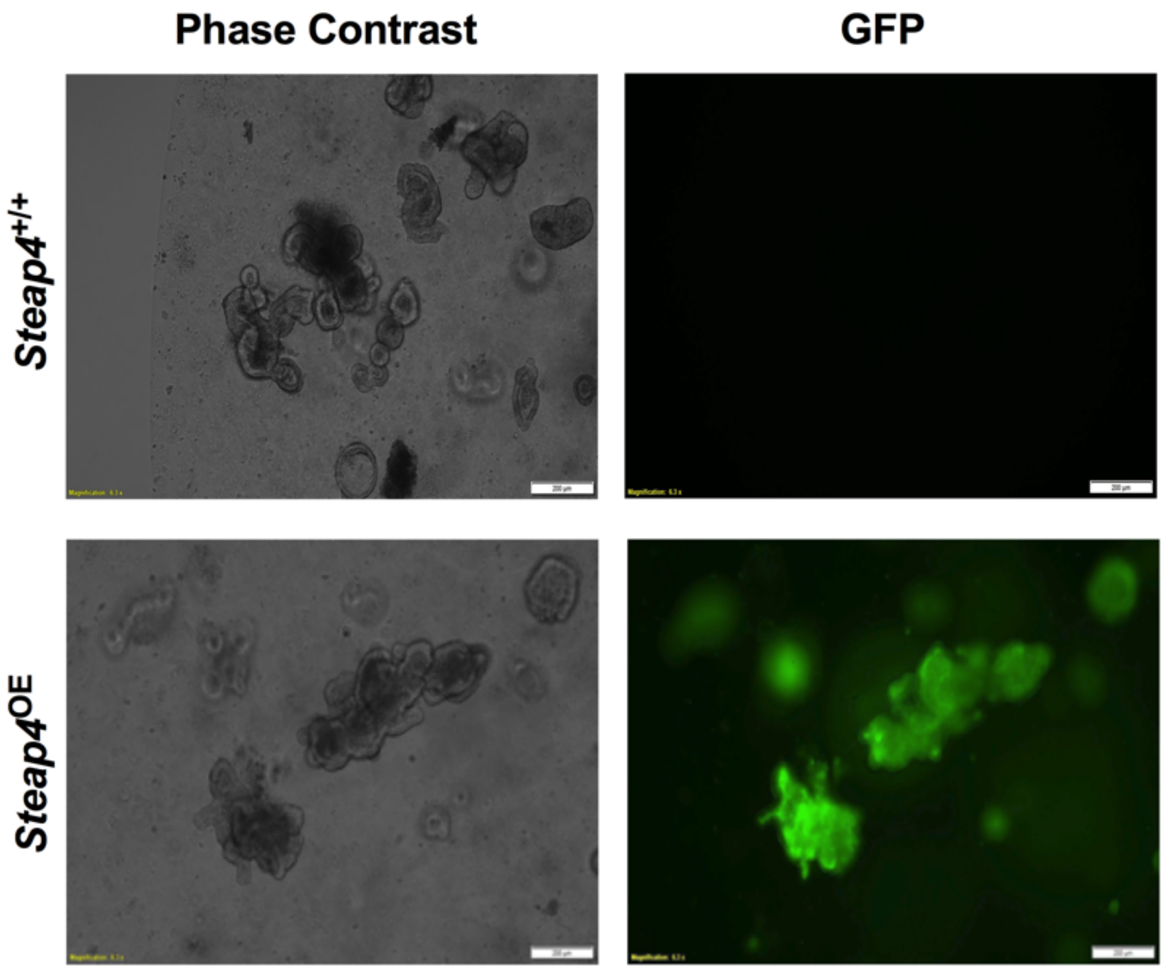

Fig. S4. Mouse colon enteroid culture derived from *STEAP4*^{OE} mice and their littermates. Phase contrast and green fluorescence (GFP) imaging for mouse enteroids passaged 3 generations and maintained in Matrigel with advanced DMEM/F12 complete media.

Journal of Cell Science • Supplementary information

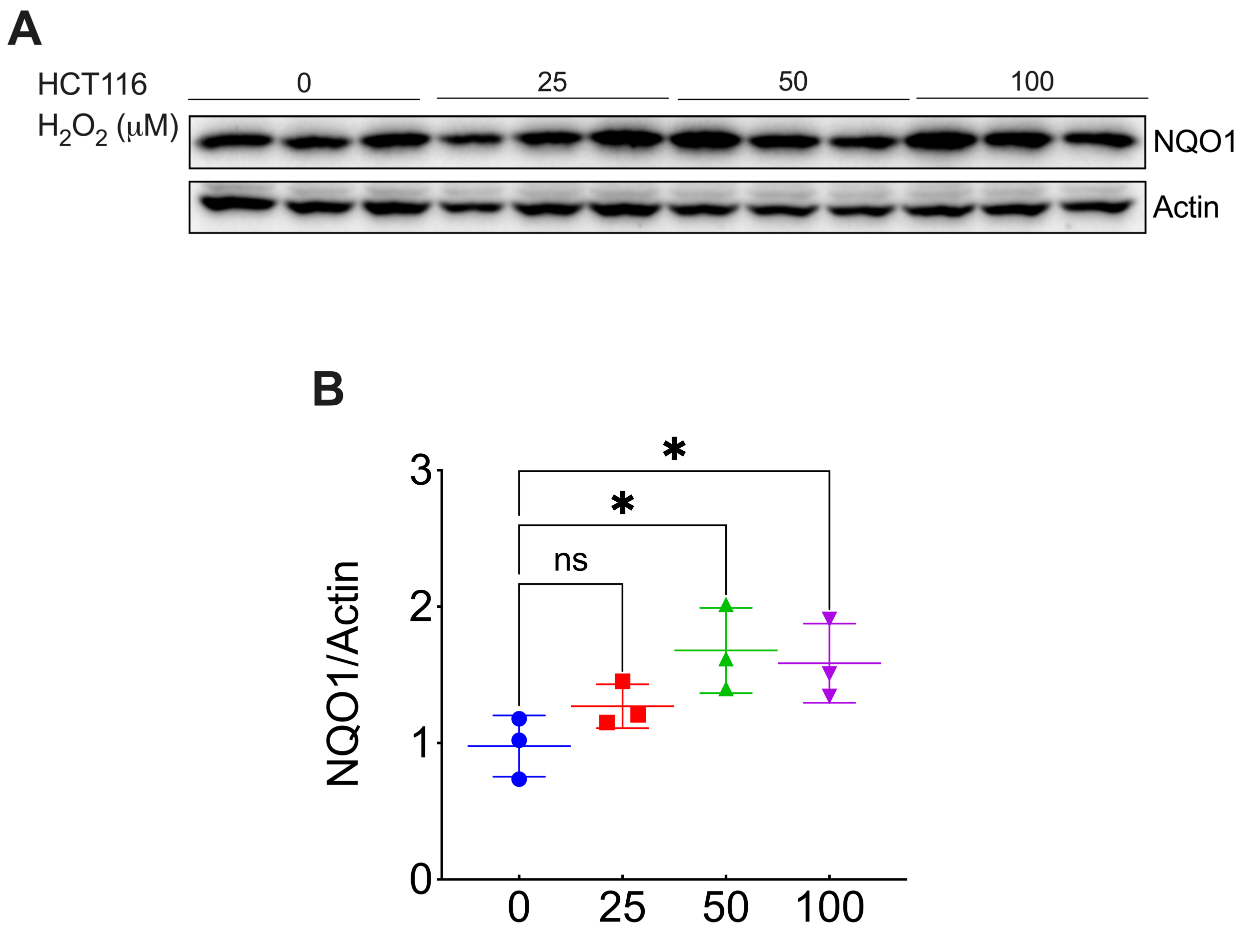

Fig. S5. H_2O_2 treatment induces NQO1 in HCT116 cells. (**A**) Western blot analysis and (**B**) quantification of NQO1 expression levels in HCT116 cells treated with different H_2O_2 concentration. *$p<0.05$. Data in B were analyzed with one way ANOVA. N=3.

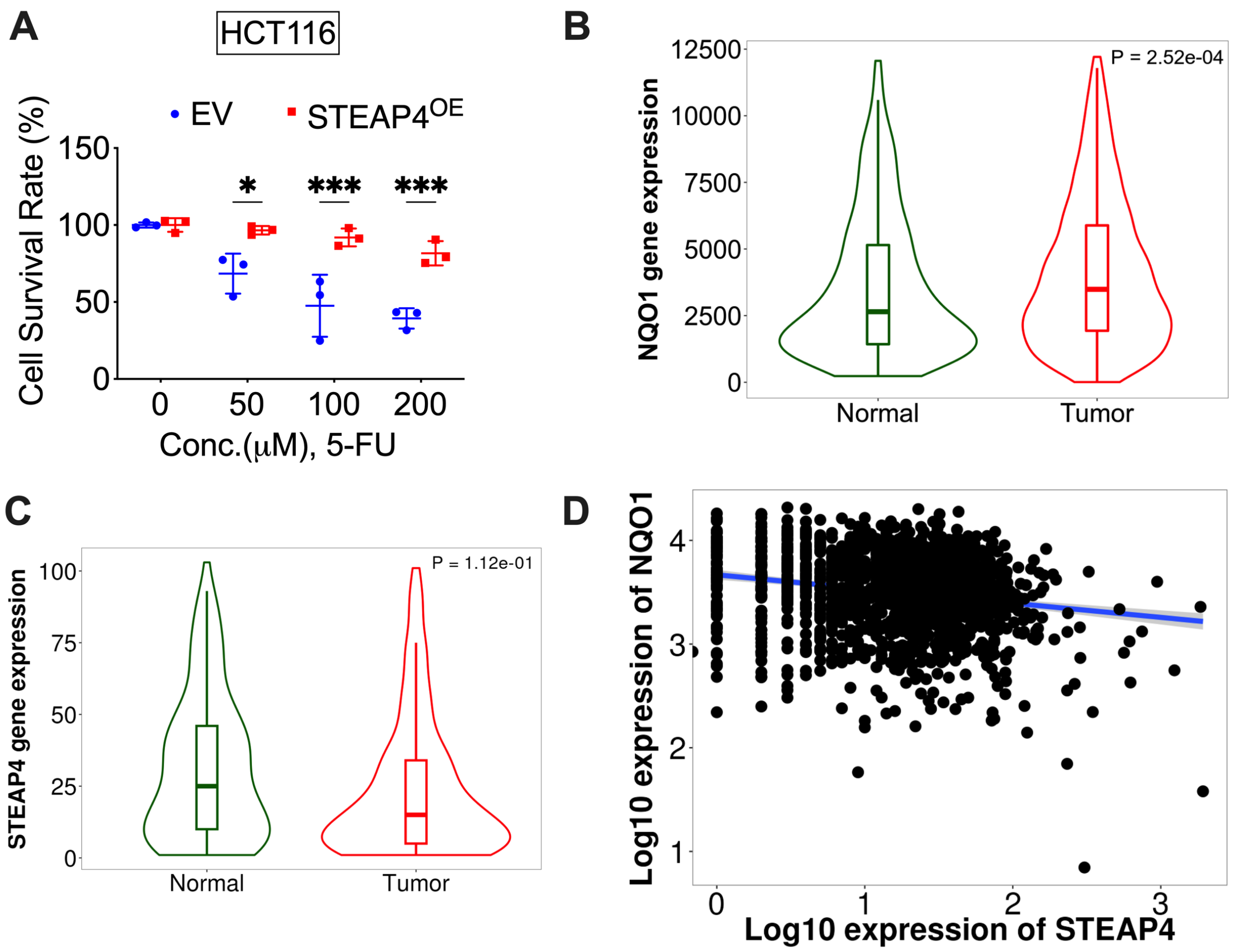

Fig. S6. **HCT116 cells with STEAP4 overexpression are resistant to 5-FU treatment**. (**A**) Cell survival rates in the HCT116 cells with or without STEAP4 overexpression treated with different concentrations of 5-FU (N=3). (**B**) NQO1 was significantly increased in colon tumors. (**C**) STEAP4 was significantly decreased in colon tumors. (**D**) NQO1 mRNA expression negatively correlates with STEAP4 in colon tumor tissues. *p<0.05, ***p<0.001. Data in A were analyzed with two-way ANOVA. Data in D were analyzed with Spearman correlation: R=-0.18, N=1450.

Journal of Cell Science • Supplementary information

Figure 2A

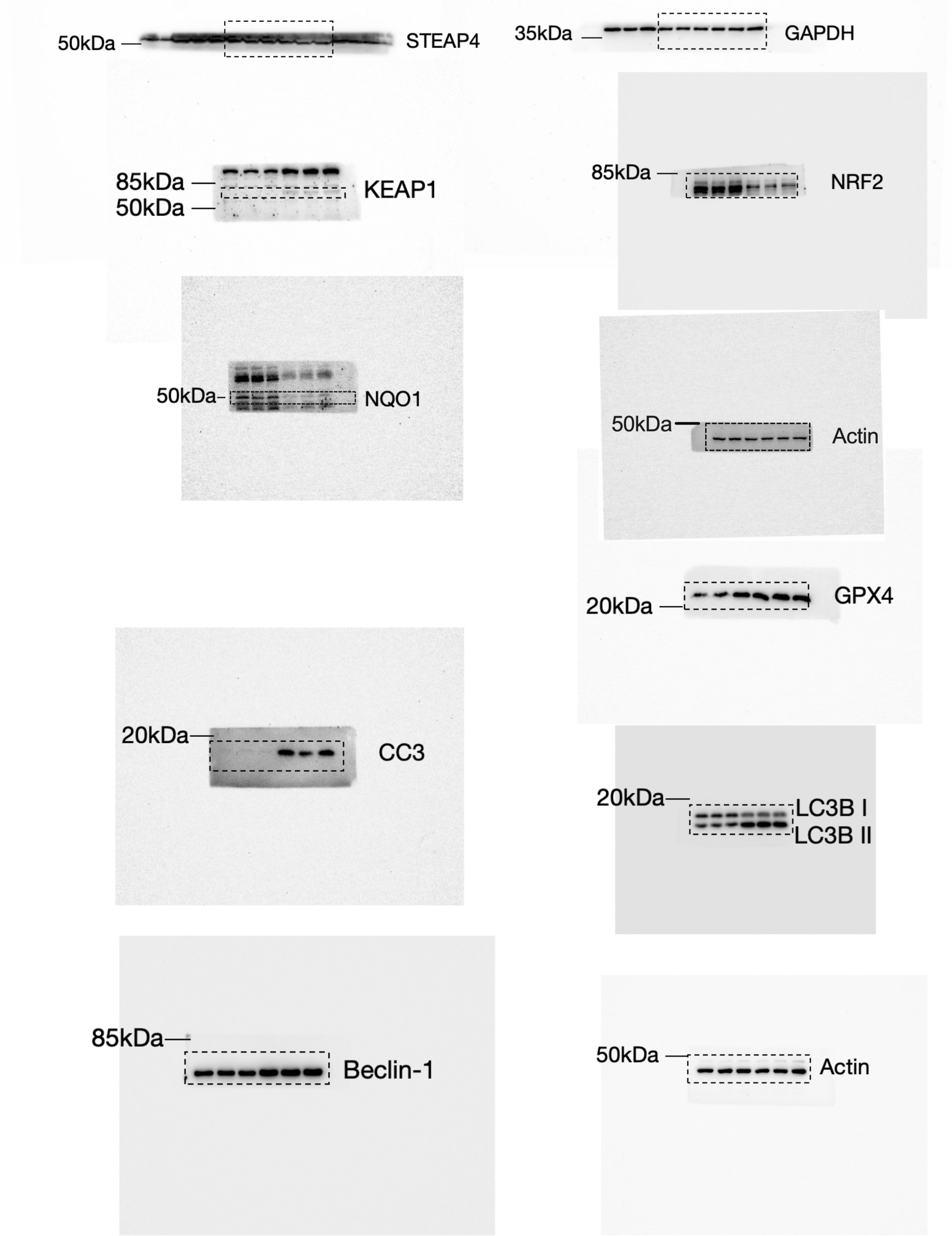

Journal of Cell Science • Supplementary information

Figure 3B

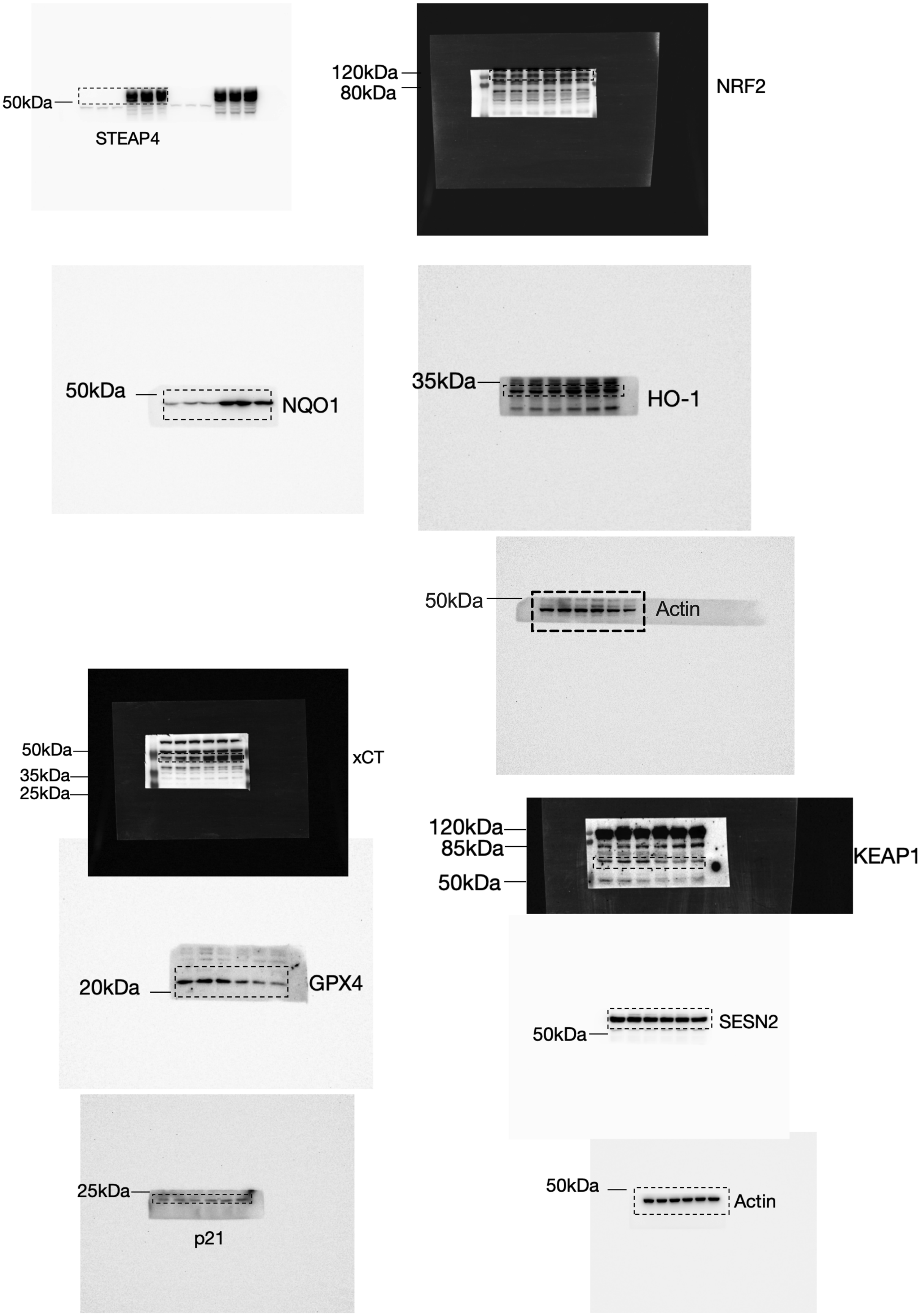

Journal of Cell Science • Supplementary information

Figure 4E

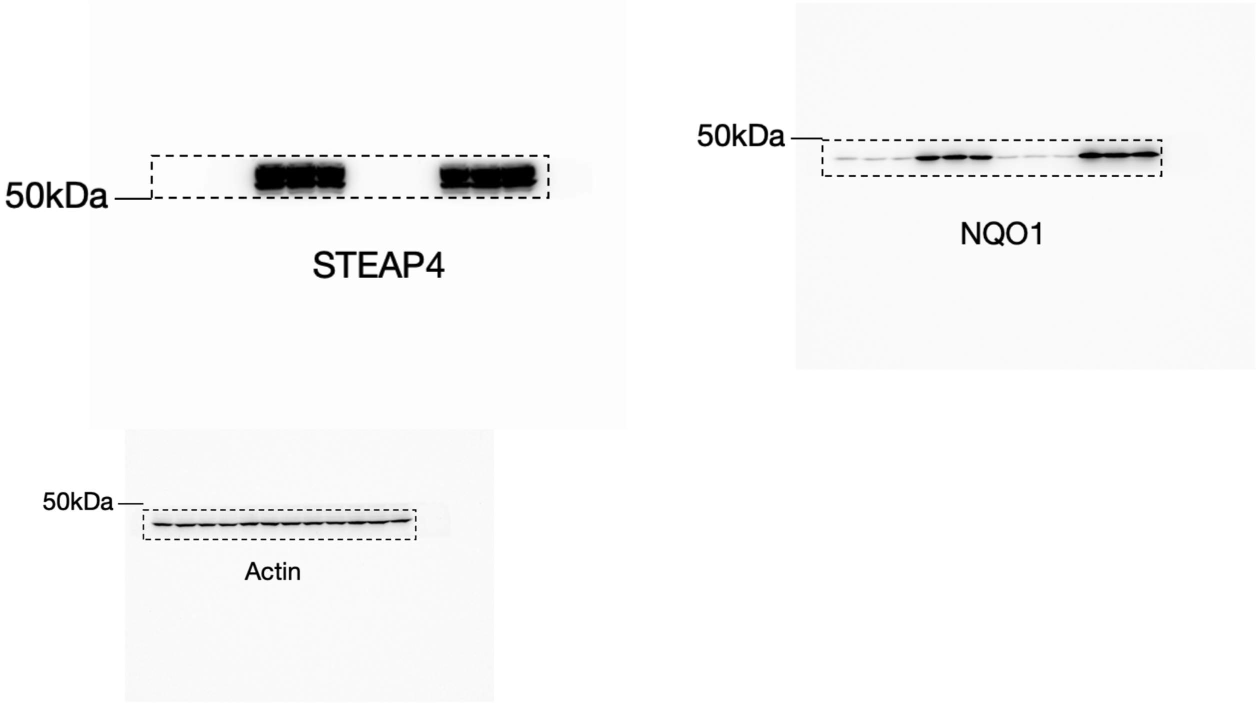

Figure 4G

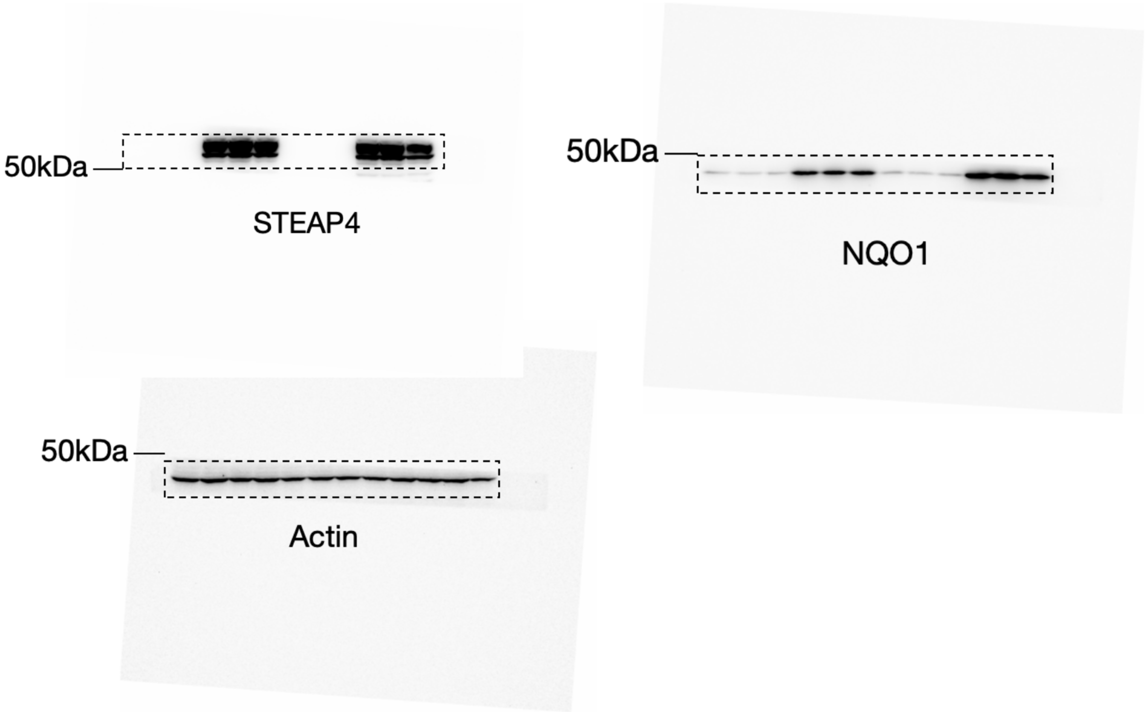

Journal of Cell Science • Supplementary information

Figure S3

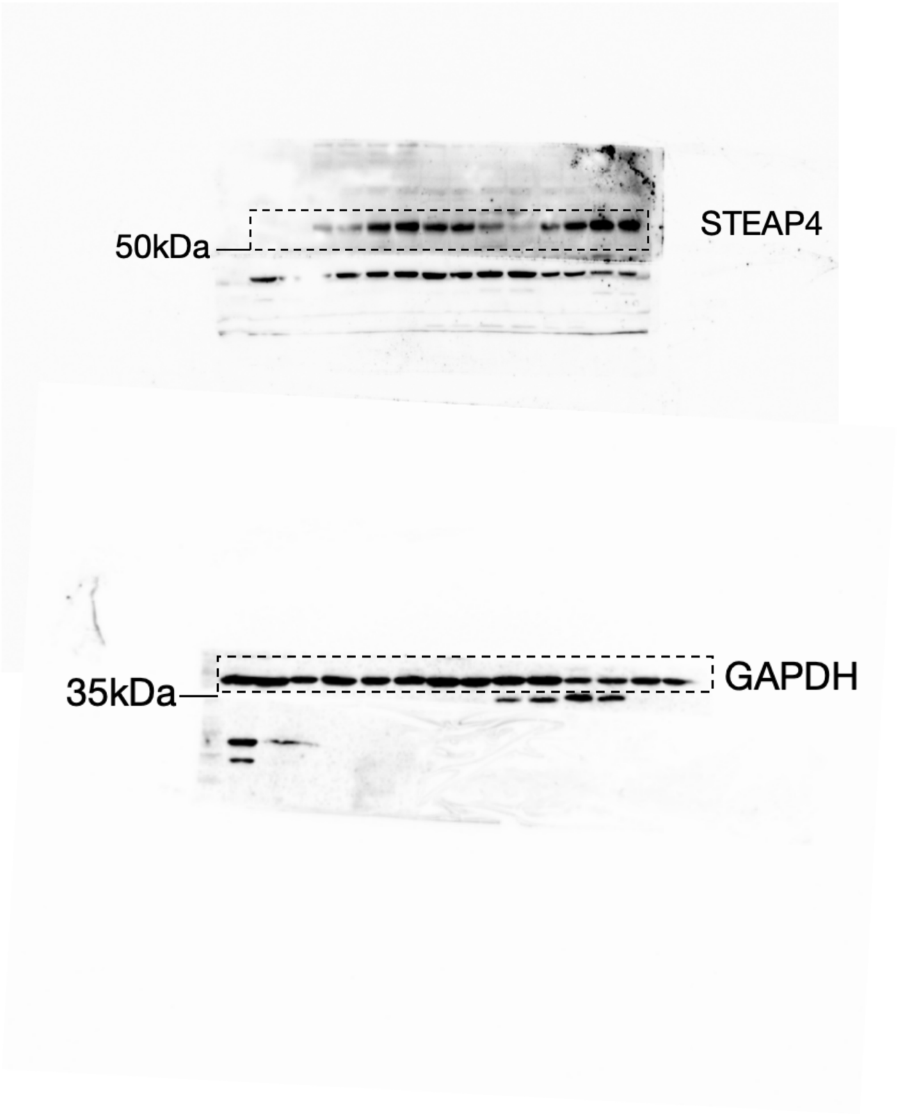

Journal of Cell Science • Supplementary information

Fig. S5

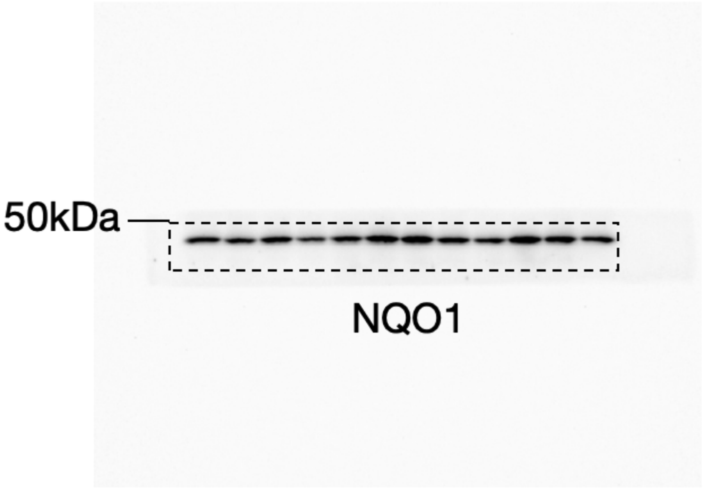

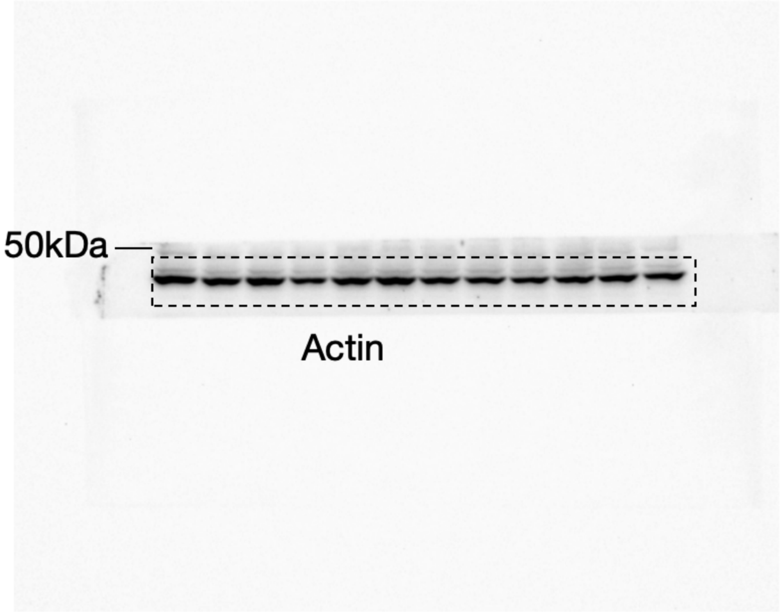

Fig. S7. Blot transparency.

Journal of Cell Science • Supplementary information

Table S1. Primers used in this study.

18s	F: GTAACCCGTTGAACCCCATT
	R: CCATCCAATCGGTAGTAGCG
Nqo1	F: CCAATCAGCGTTCGGTATTA
	R: GTCTTCTCTGAATGGGCCAG
Nrf2	F: TCTATGTCTTGCCTCCAAAGG
	R: CTCAGCATGATGGACTTGGA
Fth1	F: GGCAAAGTTCTTCAGAGCCA
	R: CATCAACCGCCAGATCAAC
Pcna	F: TTTGAGGCACGCCTGATCC
	R: GGAGACGTGAGACGAGTCCAT
Ccnd1	F: GCGTACCCTGACACCAATCTC
	R: CTCCTCTTCGCACTTCTGCTC
Steap4	F: AAAATGCAGACAACCCCTTG
	R: TGCAAGCCTAGAAGGCAGAG
Sesn2	F: TCCGAGTGCCATTCCGAGAT
	R: TCCGGGTGTAGACCCATCAC
Gpx4	F: GCCTGGATAAGTACAGGGGTT
	R: CATGCAGATCGACTAGCTGAG
Tfrc	F: TCAAGCCAGATCAGCAATTCTC
	R: AGCCAGTTTCATCTCCACATG
Ncoa4	F: GAAAAGAGGCTATATCCAGGTGC
	R: GTCCAGTGTGGGAACAGGTC
Dmt1	F: TTGGCAATCATTGGTTCTGA
	R: CTTCCGCAAGCCATATTTGT
NQO1	F: GGACTGCACCAGAGCCAT
	R: GCCTCCTTCATGGCATAGTT
NRF2	F: TCTTGCCTCCAAAGTATGTCAA
	R: ACACGGTCCACAGCTCATC
FTH1	F: GGCAAAGTTCTTCAAAGCCA
	R: CATCAACCGCCAGATCAAC
PCNA	F: ACACTAAGGGCCGAAGATAACG
	R: ACAGCATCTCCAATATGGCTGA
SESN2	F: GACCATGGCTACTCGCTGAT
	R: GCTGCCTGGAACTTCTCATC
NCOA4	F: GCAAATATTGGGCCCTTCCT
	R: CAATACCGGATGCTGACTTCTG
STEAP4	F: TCAATCCCAGTGATCTTCCA
	R: CTGGACGCTTCCTCCTTG

Journal of Cell Science • Supplementary information
